# The role of rurality and area-level disadvantage in lifestyle risk factors and psychological well-being among cancer survivors: findings from the 2021 NCI HINTS-SEER

**DOI:** 10.1007/s10552-026-02212-6

**Published:** 2026-07-09

**Authors:** Anita M. Kumar, Carrie A. Miller, Reyna Han, D. Jeremy Barsell, Joseph Boyle, Bernard F. Fuemmeler

**Affiliations:** 1 Department of Social Behavioral Sciences, IRACDA Postdoctoral Research Fellow, Virginia Commonwealth University, 830 E. Main Street, Richmond, VA 23286 USA; 2 Department of Family Medicine & Population Health, Virginia Commonwealth University, Richmond, VA USA; 3 Virginia Commonwealth University School of Medicine, Richmond, VA USA; 4 Massey Comprehensive Cancer Center, Richmond, VA USA

**Keywords:** Lifestyle health behaviors, Area-level disadvantage, Psychological well-being, Cancer survivorship, Rural–urban differences

## Abstract

**Background:**

Rural cancer survivors consistently experience poorer health outcomes than their urban counterparts, yet the interplay between rurality and area-level disadvantage remains understudied. This study examines how rural versus urban residence and neighborhood disadvantage relate to obesity, smoking, and psychological well-being among cancer survivors.

**Methods:**

Data from 1,062 HINTS-SEER participants across three registries were analyzed. Logistic regression models estimated associations between area-level factors (Social Deprivation Index (SDI) and Areas of Persistent Poverty) and health outcomes, adjusting for individual sociodemographic factors. Analyses accounted for complex survey design using Taylor series linearization.

**Results:**

Urban residents showed lower odds of obesity compared with their rural counterparts (OR: 0.69; 95% CI: 0.50–0.95). In stratified analyses, area-level social deprivation demonstrated smaller statistically significant associations. Higher deprivation was associated with increased odds of obesity among urban cancer survivors (OR: 1.01; 95% CI: 1.00–1.02) and increased depression/anxiety among rural survivors (OR: 1.02; 95% CI: 1.00–1.04). Across all models, individual-level socioeconomic factors (education, income) showed stronger, more consistent protective associations than area-level indicators.

**Conclusions and implications:**

Area-level disadvantage was a contributor to rural cancer survivors’ psychological well-being. Individual socioeconomic status remains the dominant determinant of lifestyle and mental health outcomes. Cancer survivors residing in socioeconomically disadvantaged rural areas may benefit from targeted mental health intervention. Future longitudinal research incorporating census tract–level geographic measures and residential histories is needed to clarify the mechanisms linking the geographic and socioeconomic context to survivorship outcomes.

## Introduction

The number of people living with or having a history of cancer is on the rise in the United States (US) with the number of cancer survivors projected to grow from 18.1 million in January of 2022 to 22.5 million by 2032 [1]. Cancer survivors often undergo complex and intensive treatments that can affect every aspect of their lives, including physical health and psychological well-being [2]. Understanding the factors that contribute to healthy cancer survivorship is essential for developing strategies to improve survivorship outcomes and experiences.

Lifestyle behaviors have been shown to improve quality of life and reduce cancer-related symptoms, thereby improving survivorship experiences [3]. Three highly prevalent and modifiable factors include body weight (obesity), smoking, and psychological distress (particularly depression and anxiety). Within a prospective cohort study of survivors, those who adhered to cancer prevention lifestyle recommendations had a significantly lower likelihood of disease recurrence and mortality [4]. Moreover, researchers have identified obesity as being associated with an increased risk of cancer recurrence, treatment-related complications, and reduced overall survival across multiple cancer types, including breast, colorectal, and prostate cancers [5–10]. Similarly, smoking remains a significant modifiable risk factor among cancer survivors, with continued smoking linked to poorer treatment response, increased recurrence, and shortened survival, while smoking cessation improves these outcomes [11, 12]. Cancer survivors often face a wide range of mental health challenges[13, 14] and a cancer diagnosis increases the risk of common psychiatric disorders, including depression and anxiety, which in turn, can adversely affect treatment, recovery, and overall survival [13, 15]. Given the substantial role these three lifestyle behaviors (obesity, smoking, and depression/anxiety) play in survivorship outcomes [3], it is critical to understand the factors influencing them among cancer survivors.

Socioeconomic factors at the individual-level, such as education and income, are highly associated with obesity, smoking, and mental health outcomes [16, 17]. Beyond individual factors, area-level factors such as neighborhood conditions are also linked with poorer physical and mental health outcomes [18–20]. These area-level characteristics may shape obesity, smoking behaviors, and depression/anxiety through several key pathways: (1) access to material resources (e.g., availability of healthy foods versus energy-dense foods contributing to obesity), (2) healthcare access (e.g., smoking cessation programs and mental health services), (3) built environment features (e.g., walkability and opportunities for physical activity), and (4) psychosocial stressors (e.g., neighborhood safety, financial strain, and social cohesion influencing depression and anxiety). Studies have shown an association between living in low-income or disadvantaged areas and higher rates of obesity [21–24]. Moreover, the literature indicates that cancer survivors living in areas of lower socio-economic status (SES) and higher density of fast food are at a higher risk of being obese [25]. In addition, studies have shown that moving to an area that is considered to be of lower socioeconomic status (SES) increases the likelihood of adopting smoking behaviors and decreases the likelihood of quitting smoking among those who smoke [18], potentially due to higher tobacco outlet density, social norms, and reduced access to cessation resources. Beyond physical health, area-level SES also affects mental health, with lower area-level SES levels being associated with greater levels of depression and anxiety [19, 20]. These pathways highlight how area-level disadvantage may influence obesity, smoking, and psychological distress in interconnected ways.

The effects of residing in socially deprived areas are often magnified for those living in rural areas [26–34]. In comparison to those living in poor or socially deprived urban areas, individuals living in deprived rural settings are more likely to have unhealthy lifestyle behaviors and poorer physical and mental health outcomes to include all-cause mortality [26–34]. For cancer survivors, area-level factors such as poverty and social deprivation are associated with increased cancer mortality [17, 35] and negatively affect cancer survivorship outcomes, including self-reported health [36, 37], quality of life, and life expectancy, with these effects heightened for cancer survivors living in rural areas [38–40]. These associations are likely mediated in part by higher burdens of obesity and smoking and greater unmet mental health needs (depression and anxiety) in these settings. These impacts are further amplified for minoritized individuals residing in socially and economically deprived rural areas, where marginalized populations are often over-represented and experience higher cancer incidence and mortality rates [17].

While research has explored the impact of area-level factors on lifestyle behaviors and cancer outcomes separately, there is limited evidence examining how area-level deprivation and rurality jointly influence obesity, smoking, and depression/anxiety among cancer survivors, and the pathways underlying these relationships. The purpose of this study was to examine the associations between area-level factors, such as area-level deprivation and rurality, with three specific, clinically meaningful lifestyle behaviors (obesity, smoking, and psychological distress (depression and anxiety) alongside individual sociodemographic characteristics among cancer survivors who were respondents to a recent Health Information National Trends Survey–Surveillance, Epidemiology, and End Results (HINTS-SEER) survey–a population-based survey of SEER patients.

## Methods

### Ethical approval

This study utilized publicly available, de-identified data from the 2021 HINTS-SEER dataset. As this secondary analysis did not involve direct interaction with human participants, it was deemed exempt from institutional review board (IRB) approval at Virginia Commonwealth University in accordance with federal guidelines (45 CFR 46.104). All participants in the original HINTS-SEER survey provided written informed consent prior to participation, and data were collected under protocols approved by the National Cancer Institute.

### Data source

The HINTS-SEER database was developed by the National Cancer Institute (NCI) to oversample cancer survivors and includes self-reported sociodemographic, lifestyle, and behavioral information from cancer survivors participating in the Iowa, New Mexico, and Greater San Francisco Bay Area SEER registries [41]. The HINTS-SEER dataset is unique in that it links HINTS survey responses to key data elements from the SEER program cancer registries, providing additional information on participants’ cancer diagnoses. Stratification of sampling frames was performed based on years since diagnosis and race/ethnicity, excluding nonmalignant tumors and non-melanoma skin cancers. Once consent was received, participants were selected from the sampling frame and were sent a self-administered postal questionnaire [41]. HINTS-SEER (2021), collected between January and August 2021, includes a total of 1,234 responses [41]. The overall response rate was 12.6% with very little demographic difference between those who responded and the sample frame population in each registry [41]. Of the 1,234 participants, 172 observations were removed due to missing data for at least one variable; therefore, the analytic dataset includes *n* = 1,062 participants.

### Sociodemographic variables

#### Demographic characteristics

Participant age (years), gender (male or female), and race/ethnicity (Non-Hispanic White or Other) were included in the analyses.

#### Income

Pretax household income was self-reported by HINTS-SEER participants and included as a categorical variable with four levels: $0–$19,999, $20,000–$49,999, $50,000–$99,999, and $100,000 and up. HINTS-SEER categories were combined from $0–9,999, $10,000–$14,999, $15,000 to $19,999, $20,000 to $34,999, $35,000 to $49,999, $50,000 to $74,999, $75,000 to $99,999, $100,000 to $199,999, and $200,000 and up to facilitate meaningful analysis.

#### Employment status

Employment status was self-reported and operationalized as a binary variable representing Employed or Unemployed.

#### Educational attainment

Highest level of educational attainment was self-reported. Levels of educational attainment were as follows: Less than high school, High School, Tech Training or Some College, and College Grad or Postgrad. HINTS-SEER categories were combined from less than 8 years, 8–11 years, 12 + years, or completed high school, post-high school training other than college, some college, college graduate, and post-graduate to facilitate meaningful analysis.

### Independent variables

#### Urban or rural residence

Rural/Urban designation was based on the patient’s county population using the 2013 Rural–Urban Continuum Codes. Urban–Rural Designations were categorized as follows: Urban (codes 1–3) or Rural (codes 4–9). The 2013 Rural–Urban Continuum Codes were combined from three metro areas and six non-metro areas to facilitate meaningful analysis [42]. Rural areas served as the reference category in all regression models.

#### Area-level disadvantage

Two measures were used to assess area-level disadvantage: the Social Deprivation Index (SDI) and the Areas of Persistent Poverty (APP) scale. The SDI is a composite measure using predetermined weighted factor loadings of seven demographic characteristics included in the American Community Survey (ACS) to quantify the socioeconomic variation in health outcomes [43]. The SDI was selected as the primary measure of area-level social deprivation due to its availability within the HINTS-SEER dataset structure at the county level. The present study used the 2019 SDI at the county level to determine the social deprivation of each participant’s county of residence [43]. The SDI was used as a continuous variable to capture the range of deprivation levels across counties. APP are counties that have maintained poverty rates of 20% or more for more than 30 years [44], as seen through the 1990 decennial Census, 2000 decennial Census, and 2021 small-area income and poverty estimates or the 2014–2018 five-year data series from the ACS [44]. The list of APP was last updated in 2024 using data from the 2022 Small Area Income and Poverty Estimates (SAIPE) released by the Census Bureau. The APP listing and HINTS-SEER dataset were merged using county Federal Information Processing Standards (FIPS) codes. The categories of this variable were as follows: Yes (is an APP) and No (is not an APP).

#### Confounding variables and variable selection

Individual-level sociodemographic variables (age, gender, race/ethnicity, income, employment status, education) were included a priori in multivariable models as potential confounders based on established literature linking these factors to lifestyle behaviors and mental health outcomes [16, 17, 19, 20].

To assess potential multicollinearity among covariates, we computed variance inflation factors (VIF). All VIF values were well below the conventional threshold of 10 and below the more conservative threshold of 5 (mean VIF = 2.06; maximum VIF = 4.32 for the highest income category), indicating that multicollinearity was not a substantive concern in our models. Although income, education, and employment are conceptually related dimensions of socioeconomic status, the empirical evidence confirms these variables are not sufficiently collinear to distort coefficient estimates, and all were retained in models given their distinct theoretical contributions as confounders in cancer survivorship research.

### Dependent variables

#### Obesity

Participant body mass index (BMI) was calculated using self-reported height and weight. Obesity was categorized as having a BMI ≥ 30, consistent with standard CDC and WHO definitions of obesity [45].

#### Smoking status

Smoking status was derived from self-reported data and dichotomized to either Current Smoker or Never/Former Smoker.

#### Anxiety and depression

Anxiety and depression were assessed using the Patient Health Questionnaire 4 (PHQ-4). The PHQ-4 total score (0–12) was derived from summing the scores of four questions asking how often a patient has felt the following symptoms over the last two weeks: feeling nervous, anxious, or on edge; not being able to stop or control worrying; feeling down, depressed or hopeless; and having little interest or pleasure in doing things [46]. The PHQ-4 has demonstrated reliability and validity for screening depression and anxiety [47]. Total scores were dichotomized to a binary variable representing Low (0–5) and Moderate/Severe (6–12) levels of participant depression and anxiety, consistent with standard PHQ-4 scoring conventions [46].

### Statistical analysis

Descriptive statistics, including the frequency and percentage for categorical variables and the mean and standard deviation for continuous variables stratified by urban versus rural residency, were calculated to describe cancer survivors’ characteristics and lifestyle behaviors. Next, bivariate logistic regression models were fit to determine the associations of each explanatory variable and demographic variable with each of the three outcome variables (Obesity, Smoking Status, and Anxiety/Depression), to assess univariate associations before multivariable modeling.

Multivariable logistic regression models were used to determine how individual-level and area-level factors are associated with each of the three outcomes while controlling for demographic characteristics. Finally, multivariable logistic regression models stratified by urban/rural classification were fit to determine the effects of individual and area-level factors in urban and rural areas separately, allowing us to examine potential differential effects by geographic context.

The HINTS-SEER uses sampling weights to ensure valid inferences from the responding sample to their respective population, correcting for nonresponse and noncoverage biases. Due to the limited number of observations in rural areas, replicate weights variance estimation was not used, as there were insufficient observations to compute jackknife standard errors. Therefore, Taylor series linearization estimation methods as provided in the HINTS-SEER documentation were used to account for the complex sample design of the HINTS-SEER dataset [41]. Stata 15.0 was used for all statistical analyses.

#### Model fit

To evaluate the adequacy of the fitted logistic regression models, we assessed goodness-of-fit using the Hosmer–Lemeshow test adapted for complex survey designs. Results indicated good model fit for the obesity model and the depression/anxiety model. The smoking model produced a significant goodness-of-fit statistic (*p* < 0.0001), likely reflecting the very low prevalence of current smoking in the analytic sample (3.9%). Because the poor fit of the smoking model—driven by sparse observations across covariate patterns—limits the reliability and interpretability of stratified estimates, smoking was excluded from the stratified multivariable models.

#### Effect modification

We evaluated effect modification by urbanicity using two sets of interaction analyses in the pooled survey-weighted logistic regression models, restricted to the two outcomes with adequate model fit (obesity and depression/anxiety). First, we included SDI × urban interaction terms to test whether the effect of area-level deprivation on each outcome differed by urban–rural status. Second, we included race × urban interaction terms to test whether race/ethnicity associations differed by urban–rural status. Adjusted Wald tests were used to evaluate the joint statistical significance of the interaction terms for each set of models.

#### Sensitivity analyses

We conducted three sets of sensitivity analyses to evaluate the robustness of findings. First, we re-fit all primary models substituting two alternative rural–urban classification measures: the Urban Influence Codes (UIC) [48] and the National Center for Health Statistics (NCHS) Urban–Rural Classification Scheme [49]. Second, we re-fit all models substituting a three-category metro-adjacency variable—distinguishing urban counties, nonmetro counties adjacent to a metropolitan area, and nonmetro counties not adjacent to a metropolitan area. Third, to relax the assumption of linearity in the log-odds for the SDI, we re-fit all models using a dichotomized SDI variable (counties classified as ‘Deprived’ or ‘Not Deprived’ based on the median county-level SDI distribution by state). Fourth, to assess whether the simultaneous inclusion of SDI and APP in primary models introduced collinearity bias, we re-estimated all primary multivariable models for obesity and depression/anxiety including SDI alone (without APP) and APP alone (without SDI) in separate model specifications.

## Results

Table 1 presents the study population characteristics stratified by urban (*n* = 809) and rural (*n* = 253) residence. Urban cancer survivors were more highly educated (63.4% with a college degree or higher vs. 42.3% in rural areas) and had higher household incomes (39.8% with income ≥ $100,000 vs. 25.7% in rural areas). Rural cancer survivors had higher rates of obesity (38.3% vs. 25.3%), depression/anxiety (8.7% vs. 5.8%), and residence in Areas of Persistent Poverty (7.5% vs. 4.3%). The mean Social Deprivation Index score was modestly higher among urban participants (44.5 vs. 38.3). Rates of current smoking were similarly low across both groups (3.8% urban vs. 4.0% rural) (Table 1).

**Table 1:** Baseline characteristics of study population stratified by urban versus rural residency

Characteristic	*n* = 1062 (100%)	
	Urban (*n* = 809)	Rural (*n* = 253)
Age (years) (mean ± SD)	70.96 (11.54)	70.01 (11.75)
Gender		
Female	434 (53.65)	127 (50.20)
Male	375 (46.35)	126 (49.80)
Race/ethnicity		
Non-hispanic white	167 (20.64)	36 (14.23)
Other	642 (79.36)	217 (85.77)
Income		
$0–$19,999	56 (6.922)	25 (9.881)
$20,000–$49,999	161 (19.90)	75 (29.64)
$50,000–$99,999	270 (33.37)	88 (34.78)
$100,000 and up	322 (39.80)	65 (25.69)
Employment status		
Unemployed	610 (75.40)	178 (70.36)
Employed	199 (24.60)	75 (29.64)
Educational attainment		
High school or less	100 (12.36)	54 (21.34)
Tech training or some	196 (24.23)	92 (36.36)
College	513 (63.41)	107 (42.29)
College grad or postgrad		
Area Variables		
Areas of persistent poverty (APP)^1^		
No	774 (95.67)	234 (92.49)
Yes	35 (4.326)	19 (7.510)
Social deprivation index^2^ (mean ± SD)	44.53 (22.27)	38.30 (31.49)
Lifestyle behaviors (outcome variables)		
Obesity^3^		
Not obese	604 (74.66)	156 (61.66)
Obese	205 (25.34)	97 (38.34)
Smoking		
Never/former smoker	778 (96.17)	243 (96.05)
Current smoker	31 (3.832)	10 (3.953)
Anxiety/depression		
Not severe	762 (94.19)	231 (91.30)
Moderate/severe	47 (5.810)	22 (8.696)

^1^Assessed using the Persistent Poverty Counties List

^2^Assessed using the Social Deprivation Index

^3^Obesity is defined as having a BMI of 30 or greater

*n* = 1,062

Table 2 presents the results from the bivariate logistic models for the three outcomes (Obesity, Smoking Status, and Anxiety/Depression). Bivariate results indicated that being male (OR = 0.72, CI: 0.53–0.98; *p *< 0.05), older in age (OR: 0.98, CI: 0.97–0.98; *p* < 0.05), having at least a college degree (OR: 0.53, CI: 0.53–0.98; *p* < 0.05), and having a household income of at least $100,000 (OR: 0.53, CI: 0.29–0.95; *p* < 0.05) significantly reduced the odds of being obese among cancer survivors. Additionally, those living in urban areas were less likely to be obese compared to cancer survivors residing in rural areas (OR: 0.59, CI: 0.42–0.81, *p* < 0.05). Individuals with household incomes of between $50,000 and $99,999 (OR: 0.26, CI: 0.10–0.65, *p* < 0.05) and those with household incomes of $100,000 or more (OR: 0.08, CI: 0.02–0.25, *p* < 0.05) were less likely to be current smokers. Survivors with college degrees or higher were less likely to have PHQ-4 scores that indicated moderate or severe depression/anxiety (OR: 0.40, CI: 0.19–0.83, *p* < 0.05). Neither area-level variable (SDI or APP) was significantly associated with any of the three outcomes in bivariate analyses (Table 2).

**Table 2:** Bivariate logistic models for lifestyle risk factors and psychological well-being among HINTS/SEER survey participants

Variables	Obesity^1^, OR (95% CI)	Smoking status, OR (95% CI)	Anxiety/depression, OR (95% CI)
Age	0.98*[0.97–0.99]	0.97*[0.95–1.00]	1.00 [0.98–1.02]
Gender (Ref = Female)			
Male	0.72*[0.53–0.98]	1.27 [0.64–2.50]	0.51*[0.28–0.94]
Race/ethnicity (Ref = non-Hisp. White)			
Others	0.96 [0.61–1.51]	1.15 [0.46–2.91]	0.74 [0.38–1.43]
Employment status (Ref = Unemployed)			
Employed	1.12 [0.78–1.61]	0.98 [0.44–2.18]	1.10 [0.64–1.89]
Education attainment (Ref = H.S./GED or less)			
Tech training or some college	1.37 [0.86–2.18]	2.19 [0.73–6.60]	0.96 [0.48–1.92]
College grad or postgrad	0.53*[0.33–0.86]	0.46 [0.15–1.46]	0.40*[0.19–0.83]
Income (Ref = Under $20,000)			
$20,000–$49,999	1.07 [0.56–2.06]	0.45 [0.17–1.16]	1.35 [0.54–3.37]
$50,000–$99,999	0.81 [0.44–1.49]	0.26*[0.10–0.65]	0.67 [0.28–1.64]
$100,000 and up	0.53*[0.29–0.95]	0.08*[0.02–0.25]	0.52 [0.20–1.31]
Urban/rural residence (Ref = Rural)			
Urban	0.59*[0.42–0.81]	0.77 [0.35–1.70]	0.60 [0.36–1.01]
Social deprivation index^2^	1.00 [0.99–1.01]	1.00 [0.99–1.02]	1.01 [1.00–1.02]
Areas of persistent poverty^3^ (Ref = No)			
Yes	1.14 [0.62–2.09]	2.43 [0.93–6.36]	1.66 [0.61–4.52]

^1^Obesity is defined as having a BMI of 30 or greater

^2^Assessed using the Social Deprivation Index

^3^Assessed using the Persistent Poverty Counties List

*n* = 1,062

^*^Denotes *p* < 0.05, **p < 0.01

Table 3 presents the results of the multivariable logistic regression on the effect of individual factors and area-level disadvantage on lifestyle risk factors and psychological well-being among study participants. Age was associated with a slight decrease in the likelihood of obesity (OR: 0.97, CI: 0.96–0.99, *p* < 0.01) and the likelihood of smoking behavior (OR: 0.96, CI: 0.94–0.98, *p* < 0.01). Those living in urban areas were less likely to be obese compared to participants residing in rural areas (OR: 0.69, CI: 0.50–0.95; *p* < 0.05). In addition, having a college degree or higher was associated with decreased likelihood of obesity (OR: 0.58, CI: 0.35–0.96, *p* < 0.05), while those with household incomes of between $50,000 and $99,999 (OR: 0.26, CI: 0.10–0.67, *p* < 0.01) and those with household incomes of $100,000 or more (OR: 0.06, CI: 0.02–0.25, *p* < 0.01) were less likely to be current smokers. Males were associated with lower odds of moderate or severe depression/anxiety (OR: 0.51, CI: 0.27–0.94, *p* < 0.05). No associations were detected between area-level variables (SDI or APP) and any of the three lifestyle behaviors (obesity, smoking status, and depression/anxiety) in the multivariable logistic regression model (Table 3).

**Table 3:** Multi-Variable Logistic Regression Model for the Effect of Individual and Area-Level Disadvantage on Lifestyle Risk Factors and Psychological Well-Being Among HINTS/SEER Survey Participants

Variables	Obesity^1^, OR (95% CI)	Smoking status, OR (95% CI)	Anxiety/depression, OR (95% CI)
Age	0.97**[0.96–0.99]	0.96**[0.94–0.98]	1.00 [0.97–1.03]
Gender (Ref = Female)			
Male	0.77 [0.55–1.06]	1.61 [0.83–3.13]	0.51*[0.27–0.94]
Race/ethnicity (Ref = non-hisp. white)			
Other	1.05 [0.66–1.70]	1.71 [0.61–4.79]	0.87 [0.38–1.98]
Employment status (Ref = Unemployed)			
Employed	0.90 [0.57–1.40]	1.14[0.47–2.78]	1.35 [0.60–3.04]
Education attainment (Ref = H.S./GED or less)			
Tech training or some College	1.29 [0.80–2.06]	2.12 [0.70–6.41]	1.02 [0.47–2.21]
College grad or postgrad	0.58* [0.35–0.96]	0.68 [0.20–2.32]	0.50 [0.21–1.17]
Income (Ref = Under $20,000)			
$20,000-$49,999	1.19 [0.59–2.42]	0.41 [0.16–1.04]	1.63 [0.70–3.81]
$50,000-$99,999	1.04 [0.54–2.00]	0.26**[0.10–0.67]	0.94 [0.39–2.24]
$100,000 and up	0.71 [0.35–1.42]	0.06**[0.02–0.25]	0.84 [0.30–2.37]
Urban/rural residence (Ref = Rural)			
Urban	0.69*[0.50–0.95]	1.22 [0.50–2.95]	0.70 [0.40–1.23]
SDI^2^	1.00 [1.00–1.01]	1.00 [0.98–1.02]	1.01 [1.00–1.02]
Areas of persistent poverty^3^ (Ref = No)			
Yes	0.67 [0.32–1.38]	1.30 [0.26–6.51]	0.84 [0.27–2.60]

^1^Obesity is defined as having a BMI of 30 or greater

^2^Assessed using the Social Deprivation Index

^3^Assessed using the Persistent Poverty Counties List

*n* = 1,062

^*^Denotes *p* < 0.05, **p < 0.01

Table 4 presents the results for obesity stratified by urban–rural residency. Having a college degree or higher was associated with decreased obesity (OR: 0.53, CI: 0.29–0.98, *p* < 0.05) among cancer survivors living in urban areas. Among urban residents, the Social Deprivation Index (SDI) was marginally associated with increased likelihood of obesity (OR: 1.01, CI: 1.00–1.02, *p* < 0.05), with a very small point estimate. Age was consistently associated with lower odds of obesity in both rural and urban areas (OR: 0.96, CI: 0.93–0.99; *p* < 0.01 and OR: 0.97, CI: 0.96–0.99; *p* < 0.01 respectively). (Table 4).

**Table 4:** Stratified multi-variable logistic regression model for the effect of individual and area-level disadvantage on obesity among HINTS/SEER survey participants residing in urban areas versus rural areas

Variables	Obesity^1^	
	Urban (*n* = 809); OR (95% CI)	Rural (*n* = 253); OR (95% CI)
Age	0.97**[0.96–0.99]	0.96**[0.93–0.99]
Gender (Ref = Female)		
Male	0.74 [0.50–1.08]	0.97 [0.54–1.74]
Race/ethnicity (Ref = Non-hispanic white)		
Racial/ethnic minority	1.08 [0.64–1.82]	0.85 [0.35–2.07]
Employment status (Ref = Unemployed)		
Employed	0.95 [0.56–1.61]	0.63 [0.29–1.38]
Education attainment (Ref = H.S./GED or less)		
Tech training or some college	1.20 [0.67–2.17]	1.70 [0.74–3.91]
College grad or postgrad	0.53*[0.29–0.98]	0.66 [0.24–1.76]
Income (Ref = Under $20,000)		
$20,000–$49,999	1.57 [0.66–3.75]	0.65 [0.23–1.86]
$50,000–$99,999	1.41 [0.63–3.15]	0.46 [0.15–1.42]
$100,000 and up	0.93 [0.39–2.22]	0.38 [0.13–1.16]
SDI^2^	1.01*[1.00–1.02]	0.99 [0.98–1.00]
APP^3^ (Ref = No)		
Yes	0.71 [0.28–1.85]	0.65 [0.21–2.06]

OR odds ratio, CI confidence interval, Ref reference, N number of observations

^1^Obesity is defined as having a BMI of 30 or greater

^2^Assessed using the Social Deprivation Index

^3^Assessed using the Persistent Poverty Counties List

^*^Denotes *p* < 0.05, **p < 0.01

Table 5 presents the results for anxiety/depression stratified by urban–rural residency. Notably, area-level social deprivation (SDI) was marginally associated with increased likelihood of moderate or severe depression/anxiety among rural cancer survivors (OR: 1.02, CI: 1.00–1.04; *p* < 0.05), though the effect size was small. While living in urban areas was associated with decreased likelihood of moderate or severe depression/anxiety (OR: 0.36, CI: 0.13–0.97, *p* < 0.05). Males living in urban areas had a lower risk of anxiety/depression (OR: 0.44, CI: 0.22–0.90, *p* < 0.05). Those living in lower household incomes ($20,000–$49,999) in urban areas had an elevated risk of anxiety and depression compared to the reference group (OR: 8.75, CI: 1.83–41.96, *p* < 0.01) (Table 5).

**Table 5:** Stratified multi-variable logistic regression model for the effect of individual and area-level disadvantage on psychological well-being among HINTS/SEER survey participants residing in urban areas versus rural areas

Variables	Anxiety/depression	
	Urban (*n* = 809); OR (95% CI)	Rural (*n* = 253); OR (95% CI)
Age	1.03 [0.99–1.07]	0.95 [0.89–1.01]
Gender (Ref = Female)		
Male	0.44*[0.22–0.90]	0.79 [0.24–2.56]
Race/ethnicity (Ref = Non-hispanic white)		
Racial/ethnic minority	0.79 [0.31–2.01]	1.54 [0.50–4.72]
Employment status (Ref = Unemployed)		
Employed	2.06 [0.78–5.43]	0.65 [0.09–4.47]
Education attainment (Ref = H.S./GED or less)		
Tech training or some college	1.03 [0.42–2.53]	1.27 [0.31–5.23]
College grad or postgrad	0.36*[0.13–0.97]	0.94 [0.19–4.80]
Income (Ref = Under $20,000)		
$20,000–$49,999	8.75**[1.83–41.96]	0.42 [0.11–1.66]
$50,000–$99,999	4.67 [0.92–23.87]	0.33 [0.09–1.26]
$100,000 and up	5.37 [0.92–31.42]	0.15 [0.02–1.14]
SDI^1^	1.00 [0.99–1.02]	1.02*[1.00–1.04]
APP^2^ (Ref = No)		
Yes	1.51 [0.33–6.92]	0.43 [0.09–2.08]

OR odds ratio, CI confidence interval, Ref reference, N number of observations

^1^Assessed using the Social Deprivation Index

^2^Assessed using the Persistent Poverty Counties List

^*^Denotes *p* < 0.05, **p < 0.01

### Effect modification and sensitivity analyses

SDI × urban interaction terms were statistically significant for obesity (OR = 1.02, 95% CI: 1.00–1.03, *p* < 0.01; Appendix Table 6, indicating that greater area-level deprivation was associated with higher odds of obesity among urban cancer survivors but not among rural cancer survivors—a finding consistent with the stratified model results in Table 4, where SDI was significantly associated with obesity in the urban stratum (OR = 1.01, 95% CI: 1.00–1.02, *p* < 0.05) but not in the rural stratum. The SDI × urban interaction was non-significant for depression/anxiety (Appendix Table 6).

Race × urban interaction terms were significant for obesity (OR = 1.02, 95% CI: 1.00–1.03, *p* < 0.01) but not statistically significant for depression/anxiety (Appendix Table 7), indicating the association between race/ethnicity and obesity differs by urban–rural status. Sensitivity analyses using two alternative rural–urban classification schemes (Urban Influence Codes and NCHS Urban–Rural Classification) and a three-category metro-adjacency variable yielded results consistent with the primary analysis across both outcomes (Appendix Table 8). Similarly, substituting a dichotomized version of the SDI for the continuous measure did not meaningfully alter any of the observed associations (Appendix Table 9). Finally, sensitivity analyses entering SDI and APP in separate model specifications—rather than simultaneously—yielded results consistent with the primary analysis for both obesity and depression/anxiety, suggesting that the null effect of APP and the marginal effects of SDI in stratified models are not attributable to collinearity between these two area-level measures.

## Discussion

This study examined the independent and combined roles of rurality and area-level disadvantage in lifestyle risk factors and psychological well-being among cancer survivors using county-level data derived from three SEER registries. The incorporation of contextual measures of area-level disadvantage (SDI and APP) provides an opportunity to evaluate structural determinants of survivorship outcomes alongside individual sociodemographic characteristics. In multivariable models for the overall sample, the SDI and APP were not significantly associated with the three lifestyle behaviors under investigation (obesity, smoking, and psychological distress (specifically depression and anxiety) when adjusted for individual-level covariates. This aligns with prior multilevel research suggesting that while neighborhood disadvantage is often correlated with adverse health outcomes, its independent contribution may diminish when individual-level socioeconomic characteristics are included in models. Indeed, studies using large cancer cohorts have demonstrated that although neighborhood deprivation can influence behaviors such as smoking and obesity, the proportion of variance explained by area-level factors is typically modest [50].

However, stratified analyses by urban/rural residence revealed differential patterns, suggesting effect modification by geographic context. Specifically, among rural cancer survivors, higher area-level social deprivation was marginally associated with increased likelihood of moderate to severe depression/anxiety. This finding is consistent with a broader literature demonstrating worse mental health outcomes among rural cancer survivors compared to their urban counterparts, even after adjusting for sociodemographic characteristics [39, 51]. In contrast, within urban settings, higher area-level social deprivation was marginally associated with increased obesity. This finding aligns with extensive epidemiologic evidence showing that individuals residing in disadvantaged environments, particularly in urban settings, are at increased risk for obesity due to factors such as limited access to healthy foods, reduced opportunities for physical activity, and built environment constraints [23, 52, 53].

### Interpretation and clinical context

Given the marginal statistical significance and small effect sizes observed for SDI associations in stratified analyses, these findings should be interpreted cautiously. While some associations reached conventional statistical significance, their magnitude suggests limited clinical or public health significance. Prior literature has consistently documented the importance of individual-level socioeconomic factors such as education and income as protective factors against lifestyle risk factors, including obesity and smoking [54]. The present findings support this literature in the overall sample; however, the added contribution of area-level factors beyond individual sociodemographic characteristics appears modest.

These results suggest that the relationship between geographic and socioeconomic context and health outcomes among cancer survivors is both complex and systematically unequal, rather than uniform or consistent across populations [55–57]. This interpretation aligns with prior literature demonstrating that social and contextual factors shape survivorship through multiple, interacting pathways. For example, research has shown that lower socioeconomic status is consistently associated with poorer cancer survival [58–60], driven by differences in stage at diagnosis, access to care, treatment quality, and comorbidity burden. Extending this perspective across the cancer continuum, evidence also highlights how social determinants, including financial toxicity, access to follow-up care, and psychosocial resources, continue to influence outcomes into survivorship, reinforcing disparities after treatment has ended [56, 61]. At the population level, geographic context further contributes to uneven cancer mortality patterns, with worse outcomes clustering in socioeconomically disadvantaged and resource-limited regions, underscoring the importance of place-based inequalities [37]. Together, these studies support the conclusion that survivorship outcomes are shaped by intersecting socioeconomic and geographic conditions, producing persistent and systematic disparities in health among cancer survivors.

One potential explanation for observed differences between urban and rural areas is differential healthcare access. Urban environments may offer cancer survivors greater access to medical and mental health services, whereas rural survivors may face barriers such as long travel distances and fewer healthcare providers [27, 62, 63]. Additionally, rural communities have greater geographic isolation and often have fewer social and economic resources, which could contribute to mental health challenges, including depression and anxiety [27]. These structural barriers may partially explain the observed association between area-level deprivation and mental health outcomes in rural settings.

### Study limitations

Several limitations warrant acknowledgment. First, this is a cross-sectional study, making it difficult to determine temporal relationships. Longitudinal data and residential histories would provide a more robust analysis, allowing for assessment of temporality and long-term effects.

Second, the HINTS-SEER pilot project included cancer survivors from only three SEER registries (Iowa, New Mexico, and the Greater San Francisco Bay Area) [41], which limits the generalizability of findings to other regions. The racial composition and urban–rural distribution of these areas are not nationally representative [41]. Third, reliance on self-reported data may introduce potential biases, particularly for sensitive topics such as smoking [64] and mental health [65], which are often underreported. Underreporting of these outcomes may have reduced statistical power to detect significant associations.

Fourth, reliance on county-level geographic measures introduces several interrelated constraints. HINTS-SEER data lack the geographic granularity of finer measures such as census tracts, which limits precision in capturing area-level social deprivation. County-level rural–urban classifications also mask substantial internal heterogeneity: counties vary enormously in geographic size, population characteristics, and built environment features, such that individuals assigned the same rural or urban designation may have markedly different lived experiences. Because rural–urban status is better conceptualized as a continuum rather than a binary construct, future research should incorporate census tract-level measures and continuous or multi-category rural–urban gradients. Additionally, because HINTS-SEER provides county-level rather than individual geocoordinates, formal assessment of spatial autocorrelation was not possible; future studies using geocoded data would allow explicit testing of spatial clustering in health outcomes. Fifth, the poor model fit of the smoking model and the very low prevalence of current smoking precluded inclusion of smoking in the stratified and interaction analyses. Future studies with larger samples or oversampling of current smokers among cancer survivors would be better positioned to examine geographic variation in smoking-related associations. Sixth, the rural analytical subsample (*n* = 253) provides limited statistical power, particularly for detecting associations of modest effect size in the stratified rural models. Non-significant findings in the rural stratum—including the SDI association with obesity—should therefore be interpreted with caution.

This study also has notable strengths, including the integration of multiple data sources to examine associations of geographic and area-level disadvantage with lifestyle risk factors and psychological well-being of cancer survivors. The HINTS-SEER database is unique in linking survey responses to cancer registry data, providing a comprehensive view of cancer-related health behaviors, knowledge, and outcomes.

## Conclusion

Given the modest associations observed between area-level social deprivation and mental health outcomes in rural cancer survivors, additional research is needed to clarify the pathways linking contextual disadvantage to survivorship lifestyle behaviors and psychological well-being. Future studies should also move beyond cross-sectional analyses to employ longitudinal designs capable of clarifying temporal relationships and cumulative effects of deprivation over the survivorship trajectory. Incorporating more granular geographic indicators, such as census tract-level measures, neighborhood asset mapping, and spatial access to healthcare facilities, would allow for more precise assessment of the contextual influences. Such multilevel modeling approaches would help determine whether area-level disadvantage amplifies individual-level vulnerability or whether strong individual socioeconomic resources buffer contextual risk. A better understanding of these mechanisms is needed to inform targeted, place-based interventions aimed at improving lifestyle behaviors and psychological outcomes among cancer survivors.

## Data Availability

The datasets generated during and/or analyzed during the current study are available from the corresponding author on reasonable request. HINTS-SEER (2021) data are available by request by filling out a restricted-use data request form (https://hints.cancer.gov/data/restricted-data.aspx) and signing a Data Use Agreement.

## Appendix

See Tables

**Table 6:** Effect modification: multi-variable logistic regression model for the interaction of area-level disadvantage and rurality on lifestyle risk factors and psychological well-being among HINTS/SEER survey participants

Variables	Obesity^1^, OR (95% CI)	Anxiety/depression, OR (95% CI)
Urban/rural residence (Ref = Rural)		
Urban	0.69*[0.50–0.95]	0.70 [0.40–1.23]
SDI^2^	1.00 [1.00–1.01]	1.01 [1.00–1.02]
Interaction of urban*SDI	1.02**[1.00–1.03]	0.99 [0.97–1.01]
Age	0.97**[0.96–0.99]	1.00 [0.97–1.03]
Gender (Ref = Female)		
Male	0.76 [0.55–1.06]	0.51**[0.27–0.94]
Race/ethnicity (Ref = non-hisp. white)		
Other	1.04 [0.65–1.67]	0.88 [0.39–1.98]
Employment status (Ref = Unemployed)		
Employed	0.87 [0.55–1.37]	1.37 [0.61–3.09]
Education attainment (Ref = H.S./GED or less)		
Tech training or some college	1.31 [0.82–2.09]	1.01 [0.47–2.20]
College grad or postgrad	0.57**[0.34–0.96]	0.50 [0.21–1.17]
Income (Ref = Under $20,000)		
$20,000–49,999	1.20 [0.59–2.42]	1.62 [0.70–3.79]
$50,000–$99,999	1.03 [0.53–1.98]	0.94 [0.40–2.24]
$100,000 and up	0.71 [0.36–1.43]	0.84 [0.30–2.36]
Areas of persistent poverty^3^ (Ref = No)		
Yes	0.70 [0.34–1.43]	0.82 [0.26–2.57]

^1^Obesity is defined as having a BMI of 30 or greater

^2^Assessed using the Social Deprivation Index

^3^Assessed using the Persistent Poverty Counties List

*n* = 1,062

^*^Denotes *p* < 0.05, ***p* < 0.01

^6^,

**Table 7:** Effect modification: multi-variable logistic regression model for the interaction of race and rurality on lifestyle risk factors and psychological well-being among HINTS/SEER survey participants

Variables	Obesity^1^, OR (95% CI)	Anxiety/depression, OR (95% CI)
Race/ethnicity (Ref = non-hisp. white)		
Other	1.04 [0.65–1.67]	0.88 [0.39–1.98]
Urban/rural residence (Ref = Rural)		
Urban	0.69*[0.50–0.95]	0.70 [0.40–1.23]
Interaction of urban*Race	1.02**[1.00–1.03]	0.99 [0.97–1.01]
Age	0.97**[0.96–0.99]	1.00 [0.97–1.03]
Gender (Ref = Female)		
Male	0.76 [0.55–1.06]	0.51**[0.27–0.94]
Employment status (Ref = Unemployed)		
Employed	0.87[0.55–1.37]	1.37[0.61–3.09]
Education attainment (Ref = H.S./GED or less)		
Tech training or some college	1.31[0.82–2.09]	1.01[0.47–2.20]
College grad or postgrad	0.57**[0.34–0.96]	0.50[0.21–1.17]
Income (Ref = Under $20,000)		
$20,000–$49,999	1.20 [0.59–2.42]	1.62 [0.70–3.79]
$50,000–$99,999	1.03 [0.53–1.98]	0.94 [0.40–2.24]
$100,000 and up	0.71 [0.36–1.43]	0.84 [0.30–2.36]
SDI^2^	1.00 [1.00–1.01]	1.01 [1.00–1.02]
Areas of persistent poverty^3^ (Ref = No)		
Yes	0.70 [0.34–1.43]	0.82 [0.26–2.57]

^1^Obesity is defined as having a BMI of 30 or greater

^2^Assessed using the Social Deprivation Index

^3^Assessed using the Persistent Poverty Counties List

*n* = 1,062

^*^Denotes *p* < 0.05, ***p* < 0.01

^7^,

**Table 8:** Sensitivity analysis: multi-variable logistic regression model using three-category metro-adjacency classification

Variables	Obesity^1^, OR (95% CI)	Anxiety/depression, OR (95% CI)
Race/ethnicity (Ref = non-hisp. white)		
Other	1.06 [0.66–1.70]	0.87 [0.38–2.00]
Urban/rural residence (Ref = Non-metro & non-metro adjacent)		
Metro	0.60**[0.38–0.96]	0.50**[0.25–0.99]
Non-metro, metro adjacent	0.78 [0.44–1.39]	0.50 [0.19–1.32]
Age	0.97***[0.96–0.99]	1.00[0.97–1.04]
Gender (Ref = Female)		
Male	0.76 [0.55–1.06]	0.50**[0.27–0.93]
Employment status (Ref = Unemployed)		
Employed	0.89 [0.57–1.40]	1.32[0.59–2.97]
Education attainment (Ref = H.S./GED or less)		
Tech training or some college	1.29 [0.81–2.07]	1.04 [0.47–2.26]
College grad or postgrad	0.58**[0.35–0.96]	0.49[0.21–1.16]
Income (Ref = Under $20,000)		
$20,000–$49,999	1.20 [0.59–2.42]	1.65 [0.70–3.87]
$50,000–$99,999	1.03 [0.54–1.99]	0.92 [0.39–2.19]
$100,000 and up	0.71 [0.35–1.42]	0.86 [0.30–2.43]
SDI^2^	1.00 [1.00–1.01]	1.01 [1.00–1.02]
Areas of persistent poverty^3^ (Ref = No)		
Yes	0.69 [0.34–1.42]	0.99 [0.33–2.98]

^1^Obesity is defined as having a BMI of 30 or greater

^2^Assessed using the Social Deprivation Index

^3^Assessed using the Persistent Poverty Counties List

*n* = 1,062

^*^Denotes *p* < 0.05, ***p* < 0.01

^8^,

**Table 9:** Sensitivity analysis: multi-variable logistic regression model for the effect of individual and area-level disadvantage (Dichotomized SDI) on lifestyle risk factors and psychological well-being among HINTS/SEER survey participants

Variables	Obesity^1^, OR (95% CI)	Anxiety/depression, OR (95% CI)
Race/ethnicity (Ref = non-hisp. white)		
Other	1.00 [0.62–1.59]	0.79 [0.36–1.74]
Urban/rural residence (Ref = Rural)		
Urban	0.73 [0.53–1.01]	0.71 [0.42–1.21]
Age	0.97***[0.96–0.99]	1.00 [0.97–1.04]
Gender (Ref = Female)		
Male	0.76 [0.55–1.06]	0.51**[0.27–0.94]
Employment status (Ref = Unemployed)		
Employed	0.90 [0.57–1.40]	1.33 [0.60–2.97]
Education attainment (Ref = H.S./GED or less)		
Tech training or some college	1.29 [0.80–2.06]	1.02 [0.47–2.20]
College grad or postgrad	0.58**[0.35–0.97]	0.50 [0.21–1.18]
Income (Ref = Under $20,000)		
$20,000–$49,999	1.18 [0.58–2.38]	1.57 [0.66–3.73]
$50,000–$99,999	1.03 [0.53–1.97]	0.90 [0.38–2.15]
$100,000 and up	0.71 [0.35–1.41]	0.83 [0.29–2.33]
SDI^2^ (Ref = Not Deprived)		
Deprived	1.12 [0.78–1.61]	0.94 [0.52–1.70]
Areas of persistent poverty^3^ (Ref = No)		
Yes	0.77 [0.38–1.56]	1.32 [0.43–4.08]

^1^Obesity is defined as having a BMI of 30 or greater

^2^Assessed using the Social Deprivation Index

^3^Assessed using the Persistent Poverty Counties List

*n* = 1,062

^*^Denotes *p* < 0.05, ***p *< 0.01

9.

## Abbreviations

ACS: American Community Survey
APP: Areas of Persistent Poverty
FIPS: Federal Information Processing Standards
HINTS-SEER: Health Information National Trends Survey–Surveillance, Epidemiology, and End Results
IRB: Institutional Review Board
NCHS: National Center for Health Statistics
NCI: National Cancer Institute
OR: Odds Ratio
PHQ-4: Patient Health Questionnaire-4
RUCC: Rural–Urban Continuum Codes
SAIPE: Small Area Income and Poverty Estimates
SDI: Social Deprivation Index
SES: Socioeconomic Status
UIC: Urban Influence Codes
US: United States
